# First eumonostiliferous nemertean from the Nishi-Shichito Ridge, *Genrokunemertes obesa* gen. et sp. nov. (Eumonostilifera, Nemertea)

**DOI:** 10.7717/peerj.13857

**Published:** 2022-10-03

**Authors:** Natsumi Hookabe, Keita Koeda, Yoshihiro Fujiwara, Shinji Tsuchida, Rei Ueshima

**Affiliations:** 1 Department of Biological Sciences, Graduate School of Science, The University of Tokyo, Tokyo, Japan; 2 Faculty of Science, University of the Ryukyus, Nishihara, Okinawa, Japan; 3 Research Institute for Global Change (RIGC), Japan Agency for Marine-Earth Science and Technology (JAMSTEC), Yokosuka, Kanagawa, Japan

**Keywords:** Seamount, Benthic, Systematics, Phylogeny, Deep sea, Trap, Ribbon worm

## Abstract

Nemerteans are mostly marine, benthic invertebrates, inhabiting intertidal to hadal zones. Recently, they have been recognized from deep sea with environmental DNA (eDNA) metabarcoding of sediment samples whereas any records from the locations and/or the water depth have not been documented in nemertean taxonomic publications to date. It suggests that there are major gaps between deep-sea nemertean fauna observed with eDNA and taxonomic knowledge. During a research expedition in 2021, we obtained a single specimen of eumonostiliferous nemertean from the southern part of Genroku Seamount Chain, the Nishi-Shichito Ridge, where any nemertean species have never been reported. Subsequent morphological and molecular examination reveal that the species is placed in a new genus and herein described as *Genrokunemertes obesa* gen. et sp. nov. It resembles shallow-water-dwelling *Kurilonemertes* and *Typhloerstedia*, but differs from the former in lacking eyes and the latter in possessing well-developed cephalic glands and lacking accessory nerves of lateral nerve cords. In terms of genetic distances based on partial sequences of the cytochrome c oxidase subunit I gene, *G. obesa* gen. et sp. nov. is closest to Monostilifera sp. Owase collected from Japan, which is characterized by large four eyes; the COI distance is higher than commonly observed interspecific divergences in eumonostiliferans.

## Introduction

Nemerteans (or ribbon worms) are usually marine, benthic invertebrates, inhabiting intertidal to hadal depths (Chernyshev & Polyakova, 2018). Although about 1,300 species have been recognized in the phylum (Kajihara, 2017), dozens of species have been turned out to be new to science in five years (e.g., Chernyshev & Polyakova, 2018; Chernyshev & Polyakova, 2019; Hookabe et al., 2020; Hookabe, Kohtsuka & Kajihara, 2021; Kajihara, Ganaha & Kohtsuka, 2022). In particular, benthic fauna of deep-sea nemerteans at great depths exceeding 1000 m is not well understood; currently, only 19 species were reported (Chernyshev, 2013; Chernyshev & Polyakova, 2019).

Recent ecological studies using environmental DNA (eDNA) metabarcoding analysis of sediment samples have uncovered deep-sea nemertean fauna from geographic areas and/or water depths where any nemertean species have never been documented in taxonomic literature (Guardiola et al., 2016; Sinniger et al., 2016; Klunder et al., 2020; Atienza et al., 2020). Those reference sequences were mostly unavailable from public database. eDNA metabarcoding is widely used as a powerful tool accelerating monitoring of biodiversity even for large-scale studies but still requires baseline data—reference sequence data associated with an appropriate species identification—prior to ecological assessment of deep-sea biodiversity.

During a research expedition under the project ‘Development of Biodiversity Monitoring Methods for the Management of Deep-sea Marine Protected Areas’ in 2021, we obtained a single specimen of eumonostiliferous hoplonemertean from the southern part of Genroku Seamount Chain. Although this area was known to harbor diverse megafaunal populations comprising sponges, octocorals, hydrozoans, and fish (Morgan & Baco, 2021; Calder & Watling, 2021; Fujiwara et al., 2022), macrobenthic fauna inhabiting the bottom substrates were poorly studied; in fact, any nemertean species have not been reported from the area to date. In this study, we provide a description of the eumonostiliferan as a member belonging to a novel genus, characterizing the internal morphology with histological observation. The phylogenetic position among eumonostiliferans is inferred based on molecular phylogenetic analyses using partial sequences of 16S rRNA, cytochrome c oxidase subunit I, 18S rRNA, 28S rRNA, and histone H3 gene markers.

## Materials & Methods

**Figure 1:**
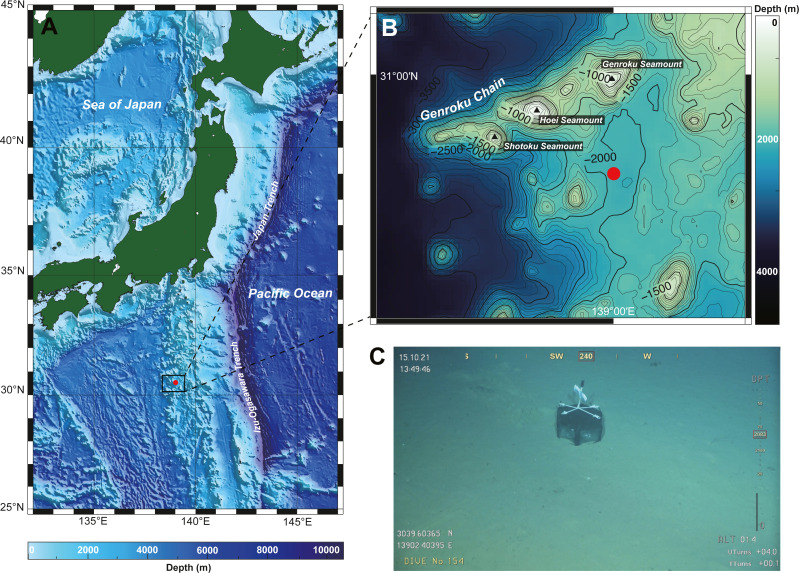
A collection site of *Gerokunemertes obesa* gen. et sp. nov. (A) Location of Genroku Seamount Chain of the Nishi-Schichito Ridge; (B) bathymetric map for Genroku Seamount Chain, a red solid circle indicating the sampling site in the present study; (C) a baited trap deployed at a depth of 2,083 m of the southern part of Genroku Seamount Chain.

A single nemertean specimen was collected from the southern part of Genroku Seamount (30°39.60′N, 139°02.41′E), on the Nishi-Shichito Ridge, Japan (Figs. 1A–1C), during a cruise of R/V *Kaimei* (cruise ID: KM21-E04C Leg1) under the research project ‘Development of Biodiversity Monitoring Methods for the Management of Deep-sea Marine Protected Areas’ in 2021. The specimen was obtained with a baited trap (handmade fish-trap: hexagonal column, 40 cm in diameter, 17 cm in height). The trap containing pieces of saury was deployed on the seafloor at a depth of 2084 m on October 13, 2021 during a remotely operated vehicle (ROV) *KM-ROV* dive #153 and was retrieved on October 15, 2021 during *KM-ROV* dive #154 by use of robotic manipulators equipped on *KM-ROV*. Photographs of the living specimen on board were taken with a digital still camera (NIKON D5600, Japan) before anaesthetization with a MgCl_2_ solution isotonic to seawater. The anaesthetized specimen was cut into two fragments by using a razor; a posterior piece (three mm in length) was preserved in 99% Ethanol (EtOH) for DNA extraction, while the remaining body for histological observation was fixed in Bouin’s fluid for 24 h, and later preserved in 70% EtOH. Serial sections were made at 7-µm thickness and stained with Mallory’s trichrome method (Gibson, 1994). Holotype has been deposited in the National Museum of Nature and Science, Tsukuba (NSMT), Japan. The electronic version of this article in Portable Document Format (PDF) will represent a published work according to the International Commission on Zoological Nomenclature (ICZN), and hence the new names contained in the electronic version are effectively published under the International Code of Zoological Nomenclature from the electronic edition alone. This published work and the nomenclatural acts it contains have been registered in ZooBank, the online registration system for the ICZN. The ZooBank LSIDs (Life Science Identifiers) can be resolved and the associated information viewed through any standard web browser by appending the LSID to the prefix http://zoobank.org/. The LSID for this publication is: urn:lsid:zoobank.org:pub:FA1B38A0-6C4F-494E-AFE4-98E4ECA5C100. The online version of this work is archived and available from the following digital repositories: PeerJ, PubMed Central SCIE and CLOCKSS”.

DNA extraction and PCR amplification were performed following Hookabe et al. (2020). After purification with EDTA/ethanol precipitation, products of terminator reactions were sent to GENEWIZ (Tokyo, Japan) for nucleotide sequencing. The newly determined sequences were deposited in the DNA Data Bank of Japan (DDBJ) under the accession numbers shown in Table 1.

**Table 1:** List of taxa included in the phylogenetic analyses with GenBank accession numbers for 16S rRNA, COI, 18S rRNA, 28S rRNA, and histone H3 genes.

Species	16S	COI	18S	28S	H3	Source
*Abyssonemertes kajiharai*	KY296888	KY296906	KY296892	KY296897	KY296915	Chernyshev & Polyakova (2018)
*Amphiporus formidabilis*	KF935498	KF935547	KF935331	KF935387	KF935443	Kvist et al. (2014)
*Amphiporus imparispinosus*	JF277618	HQ848612	JF293029	HQ856878	JF277696	Andrade et al. (2012)
*Amphiporus lactifloreus*	JF277617	HQ848611	JF293018	HQ856876	JF277672	Andrade et al. (2012)
*Antarctonemertes riesgoae*	KF935490	KF935538	KF935322	KF935378	KF935434	Kvist et al. (2014)
*Antarctonemertes unilineata*	MG920846	MG948790	–	MG920847	–	Taboada et al. (2018)
*Antarctonemertes valida*	KF935489	KF935537	KF935321	KF935377	KF935433	Kvist et al. (2014)
*Antarctonemertes* sp. Simushir	MZ231132	MZ216516	–	MZ231284	MZ216587	Chernyshev et al. (2021)
*Antarctonemertes* sp. Urup48	–	OM456697	OM423116	OM423054	OM468148	Chernyshev & Polyakova (2022)
*Argonemertes australiensis*	JF277605	HQ848601	JF293010	HQ856892	JF277750	Andrade et al. (2012)
*Asteronemertes* cf*. gibsoni*	MN211500	MN205519	MN211406	MN211456	MN205477	Chernyshev & Polyakova (2019)
*Diplomma bothwellae*	MZ231133	MZ216517	MZ231195	MZ231285	MZ216588	Chernyshev et al. (2021)
*Diplomma serpentina*	–	MN205514	MN211400	MN211450	MN205471	Chernyshev & Polyakova (2019)
Eumonostilifera sp. 2B	–	KY296910	KY296894	KY296901	KY296919	Chernyshev & Polyakova (2018)
*Galathenemertes giribeti*	MN211497	MN205516	MN211402	MN211452	MN205473	Chernyshev & Polyakova (2019)
*Genrokunemertes obesa* gen. et sp. nov.	ON244700	ON255709	ON244699	ON244696	–	Present study
*Gononemertes parasita*	JF277606	AB505821	JF304779	HQ856889	JF277745	Andrade et al. (2012) and Kajihara et al. (2011)
*Kurilonemertes dilutebasisae*	MN211494	MN205511	MN211397	MN211447	–	Chernyshev & Polyakova (2019)
*Kurilonemertes papilliformis*	MZ231131	MZ216515	MZ231194	MZ231283	–	Chernyshev et al. (2021)
*Kurilonemertes phyllospadicola*	MN211493	FJ594418 *	MN211396	MN211446	MN205468	Maslakova & Von Döhren (2009) and Chernyshev et al. (2021)
Monostilifera sp. 9 Iturup	MZ231136	MZ216521	MZ231199	MZ231289	MZ216592	Chernyshev et al. (2021)
Monostilifera sp. KuramBio2 82	–	–	MN211405	MN211455	MN205476	Chernyshev & Polyakova (2019)
Monostilifera sp. Owase	OP028962	OP034708	OP028974	OP028967	–	Present study
*Nemertellina yamaokai*	AJ436797	AJ436907	AB505826	AJ436852	AJ436959	Thollesson & Norenburg (2003)
*Oerstedia dorsalis* 2 sensu Iwata	–	MZ216523	MZ231201	MZ231291	–	Chernyshev et al. (2021)
*Oerstedia oculata*	MN211495	MN205512	MN211398	MN211448	MN205469	Chernyshev & Polyakova (2019)
*Oerstedia phoresiae*	MN211496	MN205513	MN211399	MN211449	MN205470	Chernyshev & Polyakova (2019)
*Oerstedia polyorbis*	MZ231137	MZ216524	MZ231202	MZ231292	MZ216594	Chernyshev et al. (2021)
*Psudotetrastemma* sp. KB2hop25	MN211501	MN205520	MN211407	MN211457	–	Chernyshev et al. (2021)
*Tetranemertes antonina*	–	KF935534	KF935318	KF935374	KF935430	Kvist et al. (2014)
*Tetrastemma bilineatum*	MZ231151	MZ216539	MZ231217	MZ231307	MZ216609	Chernyshev et al. (2021)
*Tetrastemma* sp. IR Iturup	MZ231181	MZ216568	MZ231254	MZ231344	MZ216645	Chernyshev et al. (2021)
*Tetrastemma* sp. IT Iturup	–	MZ216569	MZ231255	MZ231345	MZ216646	Chernyshev et al. (2021)
*Psudotetrastemma* sp. KB2hop25	MN211501	MN205520	MN211407	MN211457	–	Chernyshev et al. (2021)
*Tetrastemma vittigerum*	MZ231192	MZ216585	MZ231272	MZ231362	MZ216663	Chernyshev et al. (2021)
*Vieitezia luzmurubeae*	JF277607	HQ443426	HQ443428	HQ856890	JF277746	Andrade et al. (2012)

To infer a phylogenetic position among Oerstediina, we performed molecular phylogenetic analyses based on maximum-likelihood (ML) and using a concatenated dataset (4121-bp), comprised of partial sequences of two mitochondrial [16S rRNA (16S; 400-bp), cytochrome *c* oxidase subunit I (COI; 602-bp)] and three nuclear gene markers [18S rRNA (18S; 1750-bp), 28S rRNA (28S; 1043-bp), histone H3 (H3; 326-bp)] (Table 1); for outgroup taxa, three GenBank entities of *Amphiporus* [*Amphiporus formidabilis* (Griffin, 1898), *A. imparispinosus* (Griffin, 1898), and *A. lactifloreus* (Johnston, 1828)] were used (Table 1). Prior to the concatenation, tree topology of each gene was confirmed that there are no significant discrepancies between different nucleotide markers in regard to the relevant tree topology in this study. Sequence alignment, trimming of ambiguous sites, model selection, and phylogenetic analyses were conducted following Hookabe et al. (2020).

Uncorrected pairwise genetic distances were calculated based on 635 bp of COI by MEGA ver. 7 (Kimura, 1980; Kumar, Stecher & Tamura, 2016).

## Results

### Systematics

**Table:** 

Genus ***Genrokunemertes*gen. nov.**
urn:lsid:zoobank.org:act:860F4A8D-5A96-4AEC-B89C-1D02BC48003F

### Type species *Genrokunemertes obesa* sp. nov.

**Etymology.** The generic name is a compound word, *Genroku-* (after the type locality, the southern part of Genroku Seamount Chain) and the Greek name Nemertes.

**Diagnosis.** Eumonostiliferous nemertean lacking eyes with weakly flattened, stout body. Head with anterior and posterior cephalic furrows. Body-wall longitudinal musculature anteriorly not divided. Rhynchocoel reaching to more than 3/4 of body length; rhynchocoel musculature with inner longitudinal and outer circular layers. Mid-dorsal vessel without vascular plug. Precerebral septum present. Dorso-ventral muscles well developed. Cephalic glands well developed. Submuscular glands not developed. Cerebral organs opening around precerebral septum, posteriorly running without branching, and replaced with yellow to green glands just anterior to the brain region. Lateral nerve cords with myofibrillae but no accessory nerves.

***Genrokunemertes obesa*** sp. nov.

urn:lsid:zoobank.org:act:EEE8946C-D048-45A7-AC31-3CF3EA84DC9F

**Material examined.** Holotype: NSMT-NE H-002, female, transverse sections of anterior body fragment, 5 slides, 15 October 2021, *KM-ROV* dive #154, collected at a depth of 2084 m, south of Genroku Seamount Chain (30°39.60′N, 139°02.41′E), Nishi-Shichito Ridge, Japan.

**Figure 2:**
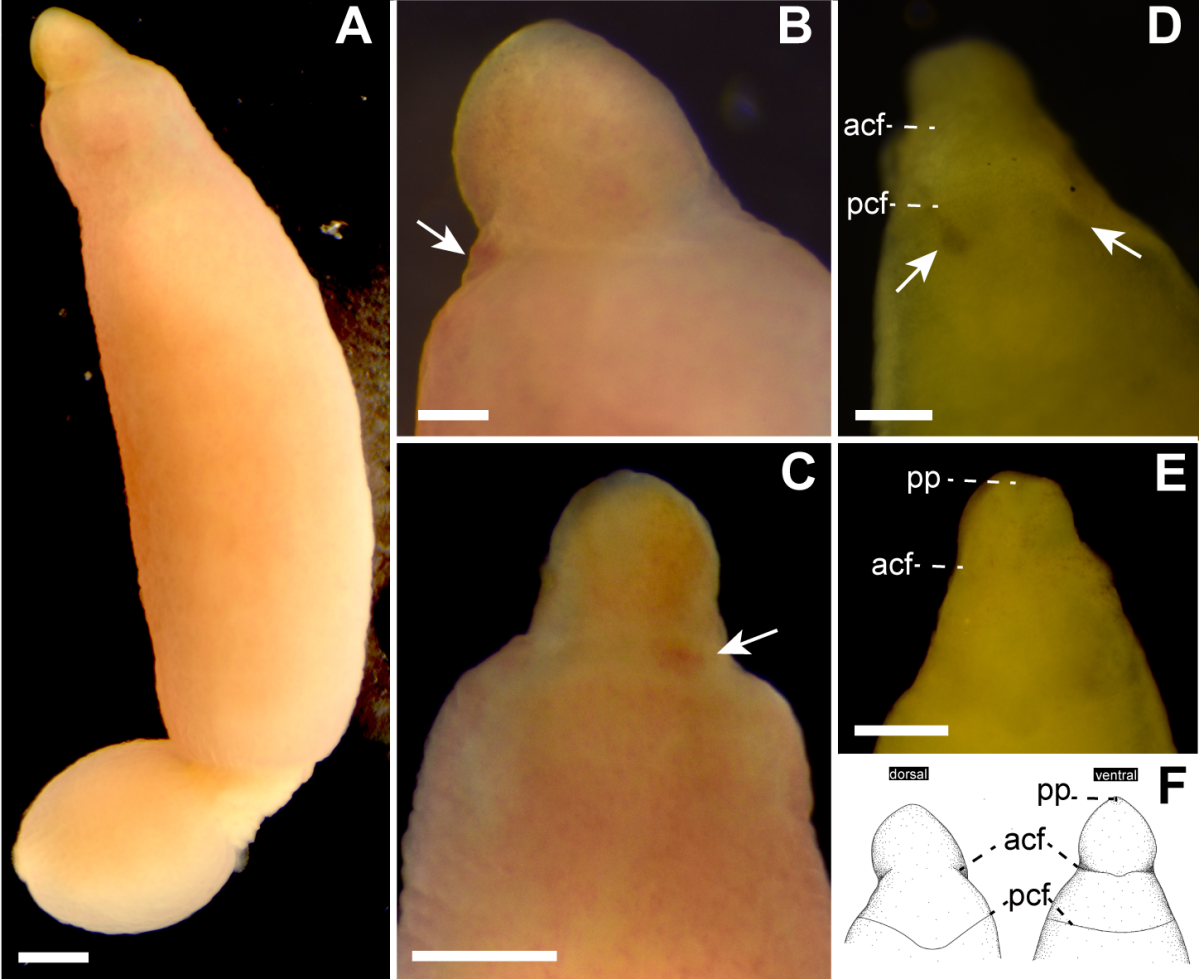
*Genrokunemertes obesa* gen. et sp. nov., holotype, NSMT-NE H-002, photographs taken in life (A–C) and after cleared with xylene (D, E), and illustrations (F). (A) Whole body, dorsal view; (B) magnification of head, living state, dorsal view, an arrow pointing to cerebral ganglia; (C) magnification of head, living state, ventral view, an arrow pointing to cerebral ganglia; (D) magnification of head, cleared in xylene, dorsal view, arrows pointing to cerebral organs; (E) magnification of head, cleared in xylene, ventral view; (F) illustrations of head, dorsal and ventral side. Abbreviations: acf, anterior cephalic furrow; pcf, posterior cephalic furrow; pp, proboscis pore. Scale bar: A, C = 500 µm; B, *D* = 250 µm.

**Description.** *External features*. Body 32.0 mm in length and 2.3 mm in maximum width; body ground color uniformly pale orange slightly with reddish tone both dorsal and ventral surfaces (Figs. 2A–2C); internal organs (intestine, gonads) visible though body wall as pale-colored region. Cerebral ganglia visible through body wall as reddish spots (Figs. 2B and 2C). Cerebral organs becoming visible thorough body wall as black spots after cleared in xylene (Fig. 2D). Cephalic furrows hardly distinguished in living specimen, but merely visible in cleared specimen in xylene (Figs. 2D and 2E); anterior cephalic furrow incompletely encircling body, opening at mid-dorsal line (Figs. 2D–2F); a single pair of posterior cephalic furrows extending posteriorly on dorsal surface and meeting each other at mid-line, ventrally forming transverse line (Fig. 2F). Eyes absent (Figs. 2B and 2D).

**Figure 3:**
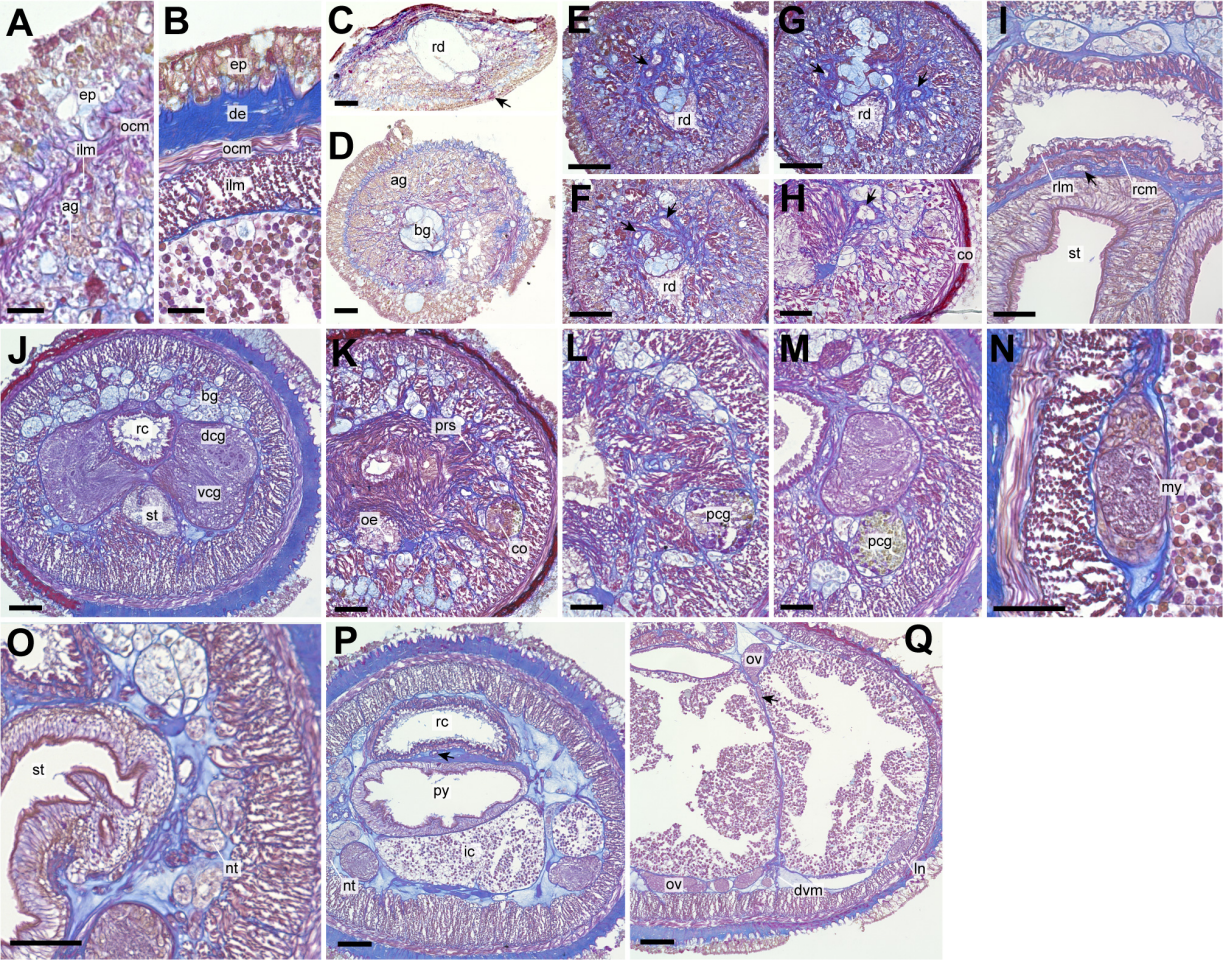
*Genrokunemertes obesa* gen. et sp. nov., holotype, NSMT-NE H-002, photomicrographs of transverse sections. (A) Body wall in precerebral region; (B) body wall in intestine region; (C) frontal organ; (D) precerebral cephalic glands; (F–H) precerebral vessels posteriorly branching above rhynchodaeum (arrow); (I) rhynchocoel wall, an arrow pointing to mid-dorsal vessel; (J) brain; (K, L) cerebral organ located anterior to brain; (M) posterior glands of cerebral organ; (N) lateral nerve cord; (O) nephridial tubules; (P) pylorus, an arrow pointing to mid-dorsal vessel; (Q) intestine, an arrow pointing to a dorsoventral process between ovary-like sacs. Abbreviations: ag, acidophilic glands; bc, basophilic glands; co, cerebral organ; de, dermis; dcg, dorsal cerebral ganglia; dvm, dorsoventral muscle; ep, epithelium; in, intestine; my, myofibril, ocm, outer circular muscle layer of body wall; oe, oesophagus, ov, ovary-like sac; pcg, posterior glands of cerebral organ; prs, precerebral septum, py, pylorus; rc, rhynchocoel; rcm, rhynchocoel outer circular muscle layer; rd, rhynchodaeum; rlm, rhynchocoel inner longitudinal muscle layer; vcg, ventral cerebral ganglia. Scale bars: A, *B* = 25 µm, C, D, N, O = 30 µm, E–H = 100 µm, I–M, P, *Q* = 50 µm.

*Internal morphology*. Epithelium 30–42 µm in thickness, with numerous red, yellow, and blue-staining gland cells and ciliated cells (Figs. 3A and 3B). Dermis up to 33 µm thick, more developed in intestinal (Fig. 3B) than precerebral region (Fig. 3A). Dorsoventral muscles between intestinal diverticula present (Fig. 3Q). Acidophilic and basophilic glands well developed in precerebral region (Fig. 3D). Submuscular glands not developed throughout the body. Cephalic lacuna posteriorly bifurcated and laterally situated at both sides of rhynchodaeum (Figs. 3E–3G). Mid-dorsal vessel without protruding into rhynchocoel (Figs. 3I and 3O). Oesophagus short, opening just posterior to precerebral septum (Fig. 3J) and leading to stomach; stomach wall gradually developed with ciliated cells and red- and yellow-stained acidophilic cells at ventral commissure of brain (Fig. 3K). Intestinal caecum anteriorly branched beneath pylorus without reaching to brain region (Fig. 3P); four pairs of branched lateral diverticula present. Proboscis lost in the specimen examined. Rhynchocoel musculature bilayered with outer circular and inner longitudinal muscle walls (Fig. 3I).

A single frontal organ present (Fig. 3C). Cerebral organ laterally opening around precerebral septum (Fig. 3H), posteriorly running without branching (Figs. 3K and 3L), and leading to yellow- to green staining glands just anterior to brain region (Fig. 3M); cerebral organ up to 105 µm in diameter. Brain with outer neurilemma (Fig. 3J); dorsal cerebral ganglia with glomerular structures (Fig. 3J). Lateral nerves with myofibrillae (Fig. 3N); an accessory nerve not found.

**Figure 4:**
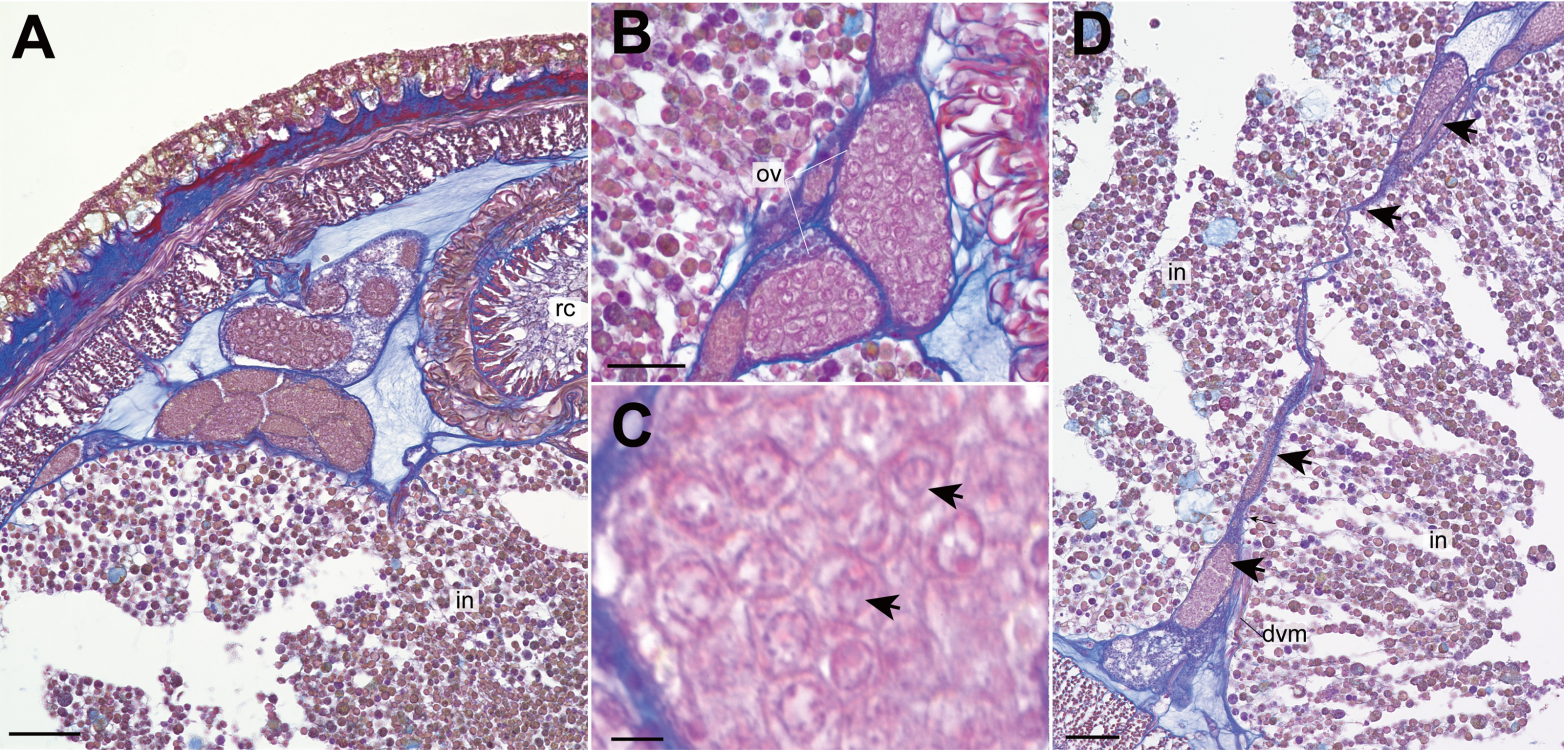
*Genrokunemertes obesa* gen. et sp. nov., holotype, NSMT-NE H-002, photomicrographs of transverse sections. (A) ovary-like sacs; (B) magnification of ovary-like sacs; (C) vesicles contained in each oocyte-like cell; (D) dorsoventral process (arrows) between ovary-like sacs. Abbreviations: dvm, dorsoventral muscle, intestine; ov, ovary-like sac; rc, rhynchocoel. Scale bars: A, *D* = 50 µm, *B* = 25 µm, *C* = 5 µm.

Nephridial tubules convoluted in posterior region of brain (Figs. 3O and 3P). Ovary-like sacs situated between inner longitudinal muscle layer and intestine (Fig. 3Q); dorsoventral processes supported by dorso-ventral muscles between ovary-like sacs (Fig. 3Q). Each ovary-like sacs containing numerous numbers of small oocyte-like cells (Fig. 4A); cells 10–13 µm in diameter; each cell containing a distinct vesicle (Figs. 4A–4C).

**Etymology.** The specific name is derived from the Latin adjective “obesus” (stout, plump), referring to stout body of the new species.

**Type locality and distribution.** The species is only known from the type locality, off south of Genroku Seamount Chain, Nishi-Shichito Ridge, Japan, at a depth of 2,084 m (Fig. 1).

**Remarks.** The present species is morphologically most similar to species in *Kurilonemertes* (Chernyshev, 1993) due to the following characters: (*i*) cylindrical and weakly flattened body, (*ii*) well-developed anterior cephalic furrows, (*iii*) body-wall longitudinal musculature anteriorly without divided, (*iv*) a single vascular plug originated from mid-dorsal vessel lacking. However, it is differentiated from *Kurilonemertes* in the lack of eyes as well as the absence of accessory nerves in lateral nerve cords; currently known three species in the genus, *Kurilonemertes papilliformis* (Korotkevitsch, 1977), *K. phyllospadicola* (Stricker, 1985), and *K. dilutebasisae* (Kulikova, 1987), possess four irregular shaped eyes (Chernyshev, 1999). Having a weakly flattened body and lacking eyes, the present species resembles *Typhloerstedia* (Chernyshev, 1999), harboring a single species *T. anadonae* (Chernyshev, 1999) (originally reported as *Oerstedia vittata* Hubrecht, 1879 from intertidal zone in Morocco (Anadon & Bitar, 1992)); however, *G. obesa* gen. et sp. nov. is differentiated from *Typhloerstedia* in having well-developed anterior cephalic furrows, cephalic glands, and cerebral organs anterior to the brain region.

### Phylogeny and genetic distances

**Figure 5:**
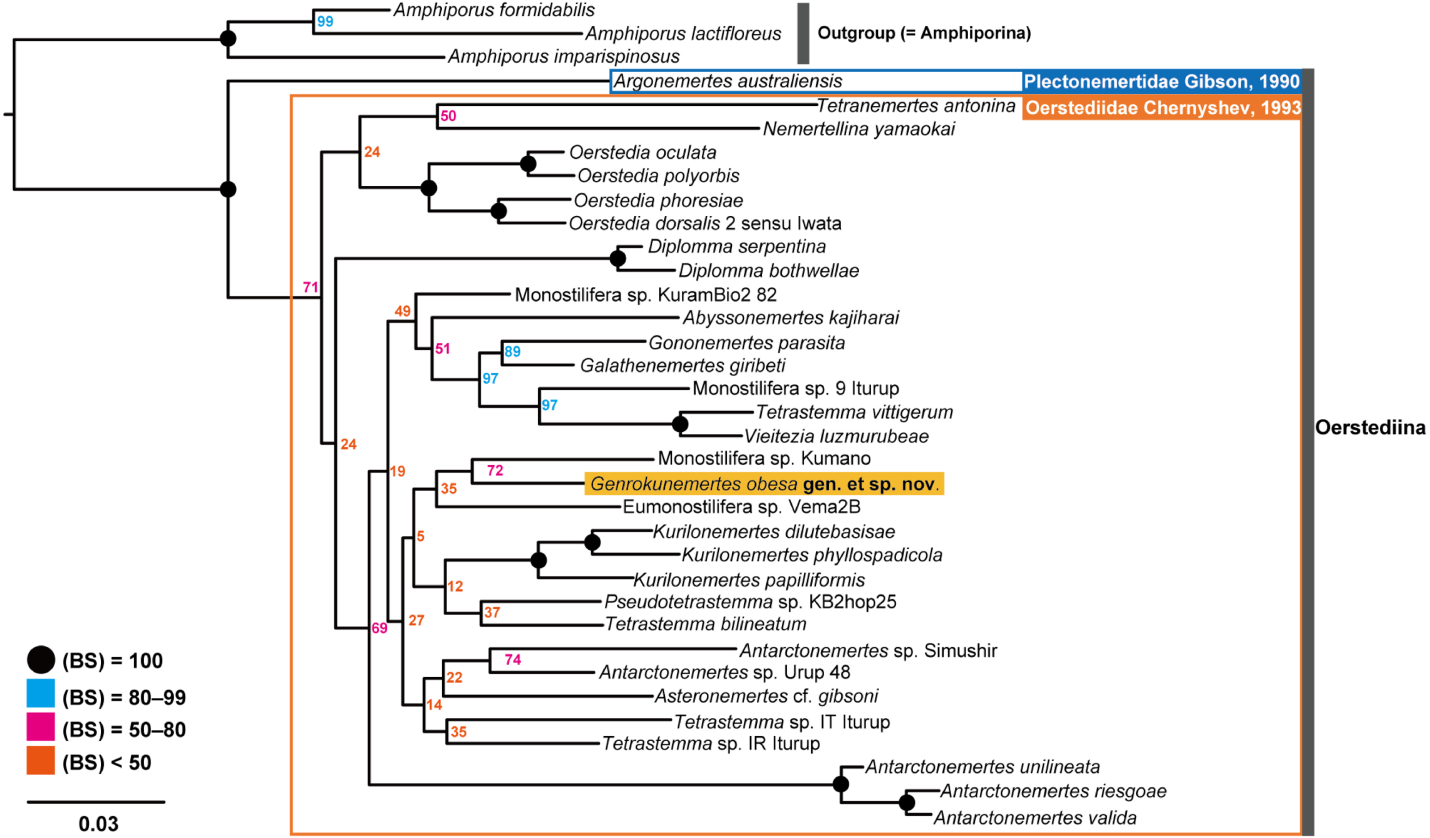
Molecular phylogenetic tree reconstructed with ML analyses using concatenated sequences of COI, 16S, 18S, 28S, and H3. Numbers near each node are bootstrap support values (BS). Solid black circles indicate a full support value, 100% of BS.

In the resulting ML tree (Fig. 5), species in Oerstediidae (Chernyshev, 1993) was sister to *Argonemertes australiensis* (Dendy, 1892), which belongs to Plectonemertidae (Gibson, 1990). Within the clade of Oerstediidae, monophyletic clades were confirmed with high support values for the following genera: *Antarctonemertes* (Friedrich, 1955) with 100% of BS, *Diplomma* (Stimpson, 1857) with 100% of BS, *Kurilonemertes* (Chernyshev, 1993) with 100% of BS, and *Oerstedia* (Quatrefages, 1846) with 100% of BS. Within a clade sister-related to *Antarctonemertes* with 69% of BS, phylogenetic relationships were not well resolved except for relationships between *Galathenemertes giribeti* (Chernyshev & Polyakova, 2019), *Gononemertes parasita* (Bergendal, 1900), *Tetrastemma vittigerum* (Bürger, 1904), *Vieitezia luzmurubeae* (Junoy, Andrade & Giribet, 2011), and Monostilifera sp. 9 Iturup (Chernyshev et al., 2021) as well as a monophyletic clade of *Kurilonemertes*. *Genrokunemertes obesa* was sister-related with Monostilifera sp. Kumano collected at depths of 150–200 m, the Sea of Kumano, Japan; the latter species possess four large eyes and a rhynchocoel as short as half of the body length (Hookabe, pers. observation).

In terms of genetic distances based on 635-bp COI sequences, *G. obesa* was genetically closer to Monostilifera sp. Kumano than all other oerstediids listed in Table 1 with 11.9% of uncorrected *p-* distance.

## Discussion

In the present study, *Genrokunemertes obesa* gen. et sp. nov. was described from the southern part of Genroku Seamount Chain at a depth of 2084 m, as the first nemertean reported from the Nishi-Shichito Ridge. It is morphologically similar to species in *Kurilonemertes* in having weakly flattened body (Fig. 2A), body-wall longitudinal musculature anteriorly without divided (Fig. 3A), and lacking a single vascular plug originated from mid-dorsal vessel, while it differs from them in lacking eyes and accessory nerves in lateral nerve cords. In our molecular phylogenetic tree, *G. obesa* was not nested within nor sister-related with the *Kurilonemertes* clade (Fig. 5). Monostilifera sp. Kumano constituted a clade with *G. obesa* with 72% of BS (Fig. 5); between the two species, 11.9% of *p*-distance based on COI (Table 2) were higher than barcoding gaps widely observed among hoplonemerteans (Sundberg, Kvist & Strand, 2016). Judging from the morphological and molecular data, the species herein described cannot be placed in currently known oerstediid genera, and thus preferred to establish a new genus for the species. As the phylogenetic position of the new taxon was not supported with a high support value possibly because of the scarcity of taxa included in our molecular phylogenetic analyses, further analyses are required based on extensive sampling including other oerstediids.

**Table 2:** Uncorrected *p-* distance (%) based on 635-bp of COI. Interspecific genetic distances between *G. obesa* and other species in Oerstediidae herein selected are given in bold.

	*G. obesa*	*K. dilutebasiae*	*K. papilliformis*	*K. phyllospadicola*	*Pseudotetrastemma* sp.	*Tetrastemma bilineatum*	Eumonostilifera sp. 2B
*Genrokunemertes obesa* gen. et sp. nov.							
*Kurilonemertes dilutebasisae*	**13.2**						
*Kurilonemertes papilliformis*	**14.5**	10.4					
*Kurilonemertes phyllospadicola*	**11.3**	9.1	10.0				
*Pseudotetrastemma* sp.	**14.3**	14.8	14.5	16.7			
*Tetrastemma bilineatum*	**13.9**	15.6	14.3	14.7	11.7		
Eumonostilifera sp. 2B	**14.3**	16.5	16.0	16.3	14.8	14.7	
Monostilifera sp. Kumano	**11.9**	15.2	14.8	13.5	12.6	13.7	13.7

Apart from the absence of eyes, *Genrokunemertes obesa* is also characterized by having sacs packed with numerous oocyte-like cells (Figs. 3Q, 3R, 4A and 4B), each of which contains a single distinct vesicle (Fig. 4C). We considered this structure to be oocytes in ovaries of this species due to: (*i*) sacs situated between intestinal caecum or diverticula as in nemertean gonads (Fig. 3Q, 3R and 4A), (*ii*) each sac surrounded by thin wall (Figs. 4A and 4B) as in hoplonemertean ovaries in the peak breeding season (Stricker et al., 2001). On the other hand, atypical features are also found in the organs—unusually small-sized oocytes compared to other hoplonemerteans (Stricker et al., 2001) and dorsoventral tube-like processes connecting dorsal and ventral ovary-like sacs (Fig. 4D). Supposedly, the two features are due to the immature state of the present specimen; immature ovaries between intestinal caecum and diverticula might appear as dorsoventral tube-like processes.

With the currently available data, we cannot exclude another possibility for the structure—body of female orthonectids interiorly packed with irregularly arranged oocytes (e.g., plasmodium of several species in *Rhopalura* (Caullery & Lavallee, 1912; Atkins, 1933)) parasitizing in nemertean ovaries although any contaminated DNA sequences were detected from total DNA extracted from the posterior body fragment containing ovary-like sacs. Further morphological studies especially on gonads and gametes with additional specimens are needed for a firm conclusion.

## Conclusions

Taxonomic studies on nemerteans dwelling deep-sea bottom are currently scarce; at depths exceeding 1000 m, only 19 species have been recognized (Chernyshev, 2013; Chernyshev & Polyakova, 2019). In the present study, we describe *Genrokunemertes obesa* gen. et. sp. nov., off south of Genroku Seamount Chain, Nishi-Shichito Ridge, as the twentieth nemertean member from the great water depth as well as the first species from the Nishi-Shichito Ridge. *Genrokunemertes obesa* gen. et sp. nov. is differentiated from morphologically close *Kurilonemertes* by the absence of eyes and *Typhloerstedia* in possessing well-developed cephalic glands and lacking accessory nerves of lateral nerve cords. The new species is also characterized by possessing sacs packed with oocyte-like cells, which are extremely smaller than typical hoplonemertean oocytes (Stricker et al., 2001). In this study, we discussed two possibilities for the peculiar structure—immature ovaries between intestinal caecum and diverticula or orthonectids parasitizing in ovaries of *G. obesa*.

##  Supplemental Information

10.7717/peerj.13857/supp-1 Supplemental Information 1 DNA sequences of *Genrokunemertes obesa* gen. et sp. nov

## Additional Information and Declarations

### DNA Deposition

The following information was supplied regarding the deposition of DNA sequences: DNA sequences of the new species herein described are available in the Supplemental Files and at GenBank: ON244696, ON244699, ON244700, ON244709.

### Data Availability

The following information was supplied regarding data availability: The data is available at GenBank: ON244696, ON244699-ON244700, ON255709, OP028962, OP028967, OP028974, and OP034708.

### New Species Registration

The following information was supplied regarding the registration of a newly described species: Publication LSID: urn:lsid:zoobank.org:pub:FA1B38A0-6C4F-494E-AFE4-98E4ECA5C100 Genus LSID: urn:lsid:zoobank.org:act:860F4A8D-5A96-4AEC-B89C-1D02BC48003F Species LSID: urn:lsid:zoobank.org:act:EEE8946C-D048-45A7-AC31-3CF3EA84DC9F
